# How a 10-minute bout requirement for physical activity affects guideline adherence estimates, Germany

**DOI:** 10.2471/BLT.25.295087

**Published:** 2026-08-20

**Authors:** Julian Brummer, Michael J Stein, Hansjörg Baurecht, Mirko Brandes, Christoph Buck, Antje Hebestreit, Lilian Krist, Michael Leitzmann, Andrea Weber, Volker Harth, Jana-Kristin Heise, Verena Katzke, Thomas Keil, Alexander Kluttig, Claudia Meinke-Franze, Rafael Mikolajczyk, Ute Mons, Katharina Nimptsch, Nadia Obi, Cara Övermöhle, Annette Peters, Tobias Pischon, Börge Schmidt, Matthias B Schulze, Henry Völzke, Stefan N Willich, Florian Herbolsheimer, Karen Steindorf

**Affiliations:** a Division of Physical Activity, Cancer Prevention and Survivorship, German Cancer Research Center, Im Neuenheimer Feld 581, Heidelberg, 69120, Germany.; b Department of Epidemiology and Preventive Medicine, University of Regensburg, Regensburg, Germany.; c Leibniz Institute for Prevention Research and Epidemiology, Bremen, Germany.; d Institute of Social Medicine, Epidemiology and Health Economics, Charité – University Medical Center Berlin, Berlin, Germany.; e Institute for Occupational and Maritime Medicine, University Medical Center Hamburg-Eppendorf, Hamburg, Germany.; f Department of Epidemiology, Helmholtz Centre for Infection Research, Braunschweig, Germany.; g Division of Cancer Epidemiology, German Cancer Research Center, Heidelberg, Germany.; h Institute for Medical Epidemiology, Biometrics, and Informatics, Medical Faculty of the Martin Luther University Halle-Wittenberg, Halle, Germany.; i Institute for Community Medicine, University Medicine Greifswald, Greifswald, Germany.; j Division of Primary Cancer Prevention, German Cancer Research Center, Heidelberg, Germany.; k Molecular Epidemiology Research Group, Max Delbrück Center for Molecular Medicine in the Helmholtz Association, Berlin, Germany.; l Institute of Epidemiology, Kiel University, Kiel, Germany.; m Institute of Epidemiology, Helmholtz Zentrum München – German Research Center for Environmental Health (GmbH), Neuherberg, Germany.; n Institute for Medical Informatics, Biometry and Epidemiology, University Hospital of Essen, Essen, Germany.; o Department of Molecular Epidemiology, German Institute of Human Nutrition Potsdam-Rehbruecke, Nuthetal, Germany.

## Abstract

**Objective:**

To examine how adherence to the World Health Organization’s (WHO) physical activity guidelines changes with and without the application of a 10-minute bout requirement.

**Methods:**

We analysed accelerometry data from the baseline assessment of the German National Cohort, NAKO. Using predefined acceleration thresholds, we classified each participant’s volumes of moderate and vigorous intensity physical activity and compared these values with WHO guidelines. We analysed data with and without the 10-minute bout requirement. We used Welch’s analysis of variance and equal proportions tests to compare demographic and health characteristics between participants who met the activity guidelines and those who did not.

**Findings:**

Of 63 134 participants, 22 226 (35.2%) met the guidelines with the bout requirement, while 62 532 (99.0%) met the guidelines without it. Participants who met the guidelines under each condition differed in demographic and health-related characteristics. For example, mean age was 48.6 years (SD: 13.0) among participants meeting the guidelines with the bout, 50.8 years (SD: 12.2) among those meeting only the guidelines without the bout and 60.8 years (SD: 10.4) among those meeting neither guidelines. Participants meeting the guidelines with the bout had a lower body mass index (25.2 kg/m^2^; SD: 4.0) than those meeting only the guidelines without the bout (27.0 kg/m^2^; SD: 5.1) and those meeting neither guidelines (30.6 kg/m^2^; SD: 7.1)

**Conclusion:**

Removing the 10-minute bout requirement for accelerometry-measured activity volumes substantially increased estimated guideline adherence. The change has implications for interpreting time trends in guideline adherence and alters conclusions about which population subgroups meet the guidelines.

## Résumé

**Objectif:**

Examiner comment le suivi des recommandations de l’Organisation mondiale de la Santé (OMS) en matière d’activité physique évolue avec et sans l’application d’une exigence de durée minimale de 10 minutes d’activité.

**Méthodes:**

Nous avons analysé les données accélérométriques issues de l’évaluation initiale de la cohorte nationale allemande (NAKO). À l’aide de seuils d’accélération prédéfinis, nous avons classé les volumes d’activité physique d’intensité modérée et élevée de chaque participant et comparé ces valeurs aux recommandations de l’OMS. Nous avons analysé les données avec et sans exigence de 10 minutes d’activité. Nous avons utilisé l’analyse de variance de Welch et des tests de proportions égales pour comparer les caractéristiques démographiques et de santé entre les participants qui suivaient les recommandations d’activité physique et ceux qui ne les suivaient pas.

**Résultats:**

Sur les 63 134 participants, 22 226 (35,2%) suivaient les recommandations ainsi que l’exigence de durée minimale d’activité, tandis que 62 532 (99,0%) suivaient les recommandations sans appliquer cette exigence. Les participants qui suivaient les recommandations dans chacune des deux conditions présentaient des caractéristiques démographiques et sanitaires différentes. Par exemple, l’âge moyen était de 48,6 ans (écart-type: 13,0) chez les participants suivant les recommandations avec exigence de durée minimale d’activité, de 50,8 ans (écart-type: 12,2) chez ceux ne suivant que les recommandations sans exigence de durée minimale d’activité et de 60,8 ans (écart-type: 10,4) chez ceux ne suivant aucune des deux recommandations. Les participants suivant les recommandations avec exigence de durée minimale d’activité présentaient un indice de masse corporelle plus faible (25,2 kg/m²; écart-type: 4,0) que ceux n’appliquant que les recommandations d’activité physique sans exigence de durée minimale (27,0 kg/m²; écart-type: 5,1) et ceux ne suivant aucune des deux recommandations (30,6 kg/m²; écart-type: 7,1).

**Conclusion:**

La suppression de l’exigence de durée de 10 minutes pour les volumes d’activité mesurés par accéléromètre a considérablement augmenté le suivi estimé des recommandations. Ce changement a des implications pour l’interprétation des tendances temporelles du respect des recommandations et modifie les conclusions concernant les sous-groupes de population qui appliquent les recommandations.

## Resumen

**Objetivo:**

Evaluar cómo cambia la adherencia a las directrices sobre actividad física de la Organización Mundial de la Salud (OMS) en función de si se aplica o no el requisito de realizar el ejercicio en sesiones de al menos 10 minutos seguidos.

**Métodos:**

Analizamos los datos de acelerometría procedentes de la evaluación inicial de la Cohorte Nacional Alemana (NAKO). Por medio de umbrales de aceleración predefinidos, clasificamos los volúmenes de actividad física de intensidad moderada y vigorosa de cada participante y comparamos dichos valores con las directrices de la OMS. Analizamos los datos con y sin el requisito de realizar ejercicio en sesiones de al menos 10 minutos seguidos. Utilizamos el análisis de la varianza de Welch y pruebas de igualdad de proporciones para comparar las características demográficas y de salud de los participantes que cumplieron las directrices de actividad física y aquellos que no lo hicieron.

**Resultados:**

De los 63 134 participantes, 22 226 (35,2%) cumplieron las directrices junto con el requisito de realizar el ejercicio físico en sesiones de al menos 10 minutos seguidos, mientras que 62 532 (99,0%) cumplieron las directrices pero sin dicho requisito adicional. En cada uno de estos dos casos fueron diferentes las características demográficas y de salud de los participantes que cumplieron con las directrices. Así por ejemplo, la edad media fue de 48,6 años (DE: 13,0) entre los participantes que cumplieron con las directrices junto con el requisito de 10 minutos seguidos de actividad física, de 50,8 años (DE: 12,2) entre aquellos que solo cumplieron las directrices pero no el requisito de las sesiones de 10 minutos de ejercicio seguidos, y de 60,8 años (DE: 10,4) entre aquellos que no cumplieron ninguno de los dos criterios. Los participantes que cumplieron con las directrices más el requisito de realizar el ejercicio en sesiones de al menos 10 minutos seguidos tenían un menor índice de masa corporal (25,2 kg/m^2^; DE: 4,0) que aquellos que solo cumplieron las directrices sin el otro requisito adicional (27,0 kg/m^2^; DE: 5,1) y que aquellos que no cumplieron ninguno de los dos criterios (30,6 kg/m^2^; DE: 7,1).

**Conclusión:**

Eliminar el requisito de que el ejercicio físico se realice en sesiones de al menos 10 minutos seguidos para los volúmenes de actividad medidos mediante la acelerometría aumentó de forma significativa la adherencia estimada a las directrices. Este cambio tiene implicaciones a la hora de interpretar las tendencias de tiempo en cuanto a adherencia a las directrices y altera las conclusiones acerca de qué subgrupos de población cumplen con dichas directrices.

## ملخص

**الغرض:**

فحص كيفية التغيّر في الالتزام بإرشادات منظمة الصحة العالمية (WHO) الخاصة بالنشاط البدني، في حالة تطبيق شرط ممارسة النشاط البدني لمدة 10 دقائق متواصلة، وحالة عدم تطبيقه.

**الطريقة:**

قمنا بتحليل بيانات قياس التسارع من التقييم الأساسي للمجموعة الوطنية الألمانية (NAKO). وباستخدام عتبات تسارع محددة مسبقًا، قمنا بتصنيف حجم النشاط البدني المعتدل والصارم لكل مشارك، وقمنا بمقارنة هذه القيم بإرشادات منظمة الصحة العالمية. كما قمنا بتحليل البيانات في حالة تطبيق شرط ممارسة النشاط البدني لمدة 10 دقائق متواصلة، وحالة عدم تطبيقه. استخدمنا تحليل التباين الخاص بـ Welch، واختبارات النسب المتساوية لمقارنة الخصائص الديموغرافية والصحية بين المشاركين الذين استوفوا إرشادات النشاط البدني والذين لم يستوفوها.

**النتائج:**

من بين 63134 مشاركًا، استوفى 22226 مشاركًا (%35.2) الإرشادات في حالة تطبيق شرط ممارسة النشاط البدني لمدة 10 دقائق متواصلة، بينما استوفى 62532 مشاركًا (%99.0) الإرشادات في حالة عدم تطبيقه. اختلفت الخصائص الديموغرافية والصحية للمشاركين الذين استوفوا الإرشادات في كل حالة. فعلى سبيل المثال، بلغ متوسط العمر 48.6 عامًا (الانحراف المعياري: 13.0) بين المشاركين الذين استوفوا إرشادات النشاط المتقطع، و50.8 عامًا (الانحراف المعياري: 12.2) بين أولئك الذين استوفوا إرشادات النشاط غير المتقطع فقط، و60.8 عامًا (الانحراف المعياري: 10.4) بين أولئك الذين لم يستوفوا أيًا من الإرشادات. وكان مؤشر كتلة الجسم لدى المشاركين الذين استوفوا إرشادات النشاط المتقطع أقل (25.2 كجم/م ^² ^؛ الانحراف المعياري: 4.0) من أولئك الذين استوفوا إرشادات النشاط غير المتقطع فقط (27.0 كجم/م ^² ^؛ الانحراف المعياري: 5.1) ومن أولئك الذين لم يستوفوا أيًا من الإرشادات (30.6 كجم/م ^² ^؛ الانحراف المعياري: 7.1)

**الاستنتاج:**

إن إلغاء شرط مدة 10 دقائق للنشاط المقاس بجهاز قياس التسارع، أدى إلى زيادة ملحوظة في الالتزام المُقدَّر بالإرشادات. هذا التغيير له آثار على تفسير الاتجاهات الزمنية في الالتزام بالمبادئ التوجيهية، ويغير الاستنتاجات المتعلقة بالفئات السكانية الفرعية التي تستوفي المبادئ التوجيهية.

## 摘要

**目的:**

检验在采用和不采用单次持续 10 分钟的要求的情况下，世卫组织 (WHO) 身体活动指南的依从率判定发生了何种改变。

**方法:**

我们分析了德国全国队列 (NAKO) 基线调查的加速度计数据。我们使用预先设定的加速度阈值，来归类每位受试者的中等强度和高强度身体活动量，然后将这些数值与世卫组织指南进行比较；我们在采用和不采用单次持续 10 分钟的要求的情况下对数据进行分析。我们使用韦尔奇方差分析与等比例检验，比较符合活动指南标准与不符合活动指南标准的受试者的人口统计学和健康特征。

**结果:**

在 63134 名受试者中，在采用单次要求的情况下，有 22226 人 (35.2%) 符合指南标准，而在不采用单次要求的情况下，有 62532 人 (99.0%) 符合指南标准。在各个情况下符合指南标准的受试者在人口统计学和健康相关特征方面有所不同。例如，在采用单次活动时间要求的情况下符合指南标准的受试者平均年龄为 48.6 岁（标准差：13.0），只在不采用单次活动时间要求的情况下才符合指南标准的受试者平均年龄为 50.8 岁（标准差：12.2），在两种情况下均不符合指南标准的受试者平均年龄为 60.8 岁（标准差：10.4）。在采用单次活动时间要求的情况下符合指南标准的受试者的身体质量指数（25.2 千克/平方米，标准差：4.0）低于在不采用单次要求的情况下符合指南标准的受试者（27.0 千克/平方米，标准差：5.1）和两种情况下均不符合指南标准的受试者（30.6 千克/平方米（标准差：7.1）。

**结论:**

针对加速度计测量的身体活动量取消单次持续 10 分钟的要求，大大提升了指南依从率的估算值。这一变化会影响对指南依从率的时间变化趋势的解读，并且改变对于哪些人群亚组能符合指南标准的结论。

## Резюме

**Цель:**

Изучить, как меняются показатели соблюдения рекомендаций Всемирной организации здравоохранения (ВОЗ) по физической активности при наличии и отсутствии требования минимальной 10-минутной активности.

**Методы:**

Были проанализированы данные акселерометрии, полученные в ходе базового обследования Немецкой национальной когорты (NAKO). Были использованы предопределенные пороговые значения ускорения, а также классифицированы объемы физической активности умеренной и высокой интенсивности каждого участника. Эти значения сравнивались с рекомендациями ВОЗ. Были проанализированы данные, полученные как с условием обязательной минимальной 10-минутной активности, так и без него. Для сравнения демографических характеристик и показателей здоровья участников, соблюдающих и не соблюдающих рекомендации по физической активности, использовались дисперсионный анализ Уэлча и критерии равенства долей.

**Результаты:**

Из 63 134 участников 22 226 человек (35,2%) соответствовали рекомендациям при учете требования к продолжительности активности, в то время как без этого требования рекомендациям соответствовали 62 532 человека (99,0%). Участники, соответствовавшие рекомендациям в каждом из сценариев, различались по демографическим характеристикам и показателям здоровья. Например, средний возраст составил 48,6 года (СО: 13,0) среди участников, выполнявших рекомендации по продолжительности активности, 50,8 года (СО: 12,2) среди тех, кто выполнял рекомендации только без учета продолжительности, и 60,8 года (СО: 10,4) среди тех, кто не соответствовал ни одному критерию. Участники, соблюдавшие рекомендации с учетом продолжительности, имели более низкий индекс массы тела (25,2 кг/м^2^; СО: 4,0), чем те, кто соответствовал только рекомендациям без учета продолжительности (27,0 кг/м^2^; СО: 5,1), и те, кто не соответствовал ни одному критерию (30,6 кг/м^2^; СО: 7,1).

**Вывод:**

Отмена требования к минимальной продолжительности физической активности (10 минут) при оценке объемов активности с помощью акселерометра существенно увеличила показатели соблюдения рекомендаций. Это изменение имеет важное значение для интерпретации временных тенденций в соблюдении рекомендаций и меняет выводы о том, какие именно подгруппы населения соответствуют этим критериям.

## Introduction

Physical activity provides many health benefits.^1^^,^^2^ Consequently, health authorities worldwide have established physical activity guidelines^1^^–^^6^ which serve as a foundation for public health strategies.^1^ In 2020, the World Health Organization (WHO) published revised physical activity guidelines.^1^^,^^4^ The revised guidelines continue to recommend a minimum of 150 minutes moderate intensity aerobic physical activity, 75 minutes of vigorous intensity aerobic physical activity or an equivalent combination of both per week.^1^ An important change, however, was the removal of the requirement that only activity bouts of at least 10 minutes could count towards the weekly goal (henceforth referred to as the bout requirement).^1^ Within the broader evolution of physical activity guidelines in recent decades,^7^^,^^8^ the bout requirement became increasingly obsolete as device-based measurements of physical activity gained prominence. This shift coincided with evidence supporting the health benefits of physical activity of any duration, which could be assessed without relying solely on participants’ self-reports.^9^^,^^10^ The implications of the change to the bout requirement on guideline adherence remain unclear.

The broader definition of guideline-relevant physical activity means that, irrespective of actual changes in population physical activity, more individuals are now likely to be classified as adhering to the guidelines. Few studies address this issue and those that do use varying methodologies and report inconsistent findings. Several accelerometry-based studies observed that removing the 10-minute bout requirement substantially increased adherence to moderate and vigorous physical activity guidelines.^11^^–^^13^ For example, a nationally representative Canadian study reported that removing the 10-minute bout requirement increased guideline adherence from 18.5% to 45.3%,^11^ while a smaller-scale Japanese study found an even greater increase from 12.5% to 92.4%.^12^ In contrast, two self-report-based studies found only marginal changes when removing the bout requirement.^14^^,^^15^ These differences in adherence after removing the bout requirement may affect subgroups of the population differently,^11^^,^^12^ which could have important implications for targeted physical activity promotion.

This study aimed to determine how accelerometry-measured adherence to the WHO aerobic physical activity guidelines changes with and without the application of the 10-minute bout requirement. We also aimed to examine the demographic and health-related characteristics of participants meeting the guideline under both conditions.

## Methods

The reporting of this study follows the STROBE guidelines.^16^

### Study setting

We used data from the baseline examination of the German National Cohort (NAKO) study, conducted between 2014 and 2019.^17^ NAKO is an ongoing multidisciplinary, multicentre, population-based prospective cohort study, whose framework, design, and methods have previously been described in detail.^17^^–^^19^ The NAKO study used random age- and sex-stratified sampling from compulsory population registries in 16 study regions, comprising 18 study centres across Germany. At baseline, the study included 205 414 participants from 1 316 055 million randomly selected eligible residents, marking a response proportion of 15.6%.^19^ Participants were aged 19–74 years at baseline.^17^ All relevant ethics committees approved the study before onset. The NAKO study follows the Declaration of Helsinki and all participants provided written informed consent. All study data can be applied for and obtained via the NAKO electronic application portal.^20^

### Physical activity assessment

A subsample from the NAKO baseline examination took part in seven-day, triaxial, hip-worn, 100Hz accelerometry using ActiGraph (Pensacola, United States of America) devices to measure physical movement.^21^ The NAKO study sampling strategy for this subsample of its multilevel data collection is described in detail elsewhere.^17^ In brief, accelerometry was assigned to 50% of participants undergoing only the basic examination programme (74 222 out of 148 444 participants).^17^ However, some study centres exceeded that proportion, resulting in 89 949 out of the 205 414 participants being offered accelerometry.^17^ As participation in the accelerometry module was voluntary, not all assigned participants took part, and the data set we received from the NAKO team contained accelerometry data from 72 713 participants.

The evolution of ActiGraph product line during the NAKO baseline examination necessitated the use of three ActiGraph models (GT3X+, wGT3X+, and wGT3X-BT).^20^ NAKO study personnel attached the ActiGraph devices to participants during the study centre visit, placing them above the right hip on the mid-axillary line using an elastic strap, wearable over or underneath clothing.^21^ The NAKO team asked participants to conduct their usual activities and remove the ActiGraph devices only during prolonged (more than 30 minutes) water exposure. Based on the methodological recommendations of the NAKO Expert Group on Physical Activity and Fitness, we considered days to be valid if the device recorded at least 16 valid hours per day. With the advice of the expert group, we evaluated the validity of any single measurement day before examining whether the required number of weekdays and weekend days was available, and excluded participants with less than one valid weekend day or fewer than two valid weekdays. Additionally, we excluded participants with an incomplete 24-hour cycle (participants with consistently invalid data for any given 15-minute interval across all recording days) to ensure full representation of the 24-hour cycle. The quality control measures and data processing of the raw accelerometry data have been described in detail elsewhere.^21^ Briefly, we primarily processed the data using GGIR (Version 2.10–3) an open-source R package;^22^ we excluded recordings with calibration errors exceeding 0.02 units of gravitational acceleration, detected and imputed non-wear time, and derived summary metrics for physical activity.^21^

We used the mean amplitude deviation to quantify time spent at different physical activity intensities.^21^ By applying established acceleration thresholds^23^ we calculated each participant’s weekly duration of moderate and vigorous intensity physical activity. In our primary analyses we applied thresholds at 90 milligravity for moderate and 410 milligravity for vigorous intensity activity (where 1 g ≈9.81 m/s2). For sensitivity analyses, we also applied lower and higher thresholds:^21^ 80 milligravity and 100 milligravity for moderate, and 400 milligravity and 420 milligravity for vigorous intensity activity. We aggregated the raw data over a time interval of five seconds, equating to an epoch length of five seconds. To derive the weekly volumes of moderate and vigorous intensity activity, we multiplied the average daily values by seven. We winsorized outliers at the 99.9th percentile within each age and sex group to address possible calibration issues or sensor malfunctions,^21^ because some extreme values persisted despite rigorous exclusion criteria.

We dichotomized adherence to the WHO physical activity guidelines, categorizing participants as adherent or non-adherent based on the following criteria: (i) at least 150 minutes per week of moderate intensity activity; (ii) at least 75 minutes per week of vigorous intensity activity; or (iii) an equivalent combination of moderate and vigorous intensity activity, calculated as minutes per week of moderate intensity plus two times minutes per week of vigorous intensity totalling at least 150 minutes per week.

We assessed guideline adherence in two ways: (i) applying the 10-minute bout requirement^11^, following the previous WHO guidelines^24^ and requiring at least 80% of epochs to meet the threshold criteria; and (ii)  removing the 10-minute bout requirement, consistent with the current WHO guidelines.^1^

We extended previous work^11^ by categorizing participants into three mutually exclusive groups: (i) adherents, who met the previous guidelines with the bout and therefore also met the current guidelines without the bout; (ii) transitioners, who met only the guidelines without the bout; and (iii) non-adherents, who did not meet either set of guidelines.

### Statistical analysis

We computed descriptive statistics, including means and standard deviations (SD). We used paired t-tests to compare volumes of moderate and vigorous intensity physical activity. To compare adherents, transitioners and non-adherents by age, body mass index (BMI), self-reported overall health (scale from one to five, from poor to excellent) and activity volumes, we applied Welch's analysis of variance followed by Games–Howell post hoc tests. We chose these procedures because Levene's tests indicated that the assumption of homogeneity of variance was violated for all variables of interest (P-values: < 0.001). To compare the three groups by sex and highest educational attainment, we used tests of equal proportions (prop.test function in R) and pairwise comparisons for proportions, with Bonferroni correction (pairwise.prop.test function in R). For educational level, we used the 2011 International Standard Classification of Education to classify participants as having low (primary and lower-secondary education), medium (upper-secondary and post-secondary non-tertiary education) or high (bachelor’s level or equivalent and above).^25^ Where indicated, we report P-values and 95% confidence intervals (CI) to evaluate statistical significance.

We used R version 4.4.1,^26^ in RStudio version 2024.04.2, (Posit Software, PBC, Boston, USA) to conduct all statistical analyses.

## Results

The analysis included data from 63 134 participants (Fig. 1). The sample had a relatively balanced sex distribution. The mean age of participants was 50.1 years (SD: 12.6; range: 19.0–74.0), with a mean BMI of 26.4 kg/m2 (SD: 4.8; range: 14.4–62.2) and they reported relatively good overall health with a mean score of 3.3 points (SD: 0.7; range: 1–5; Table 1).

**Fig. 1:**
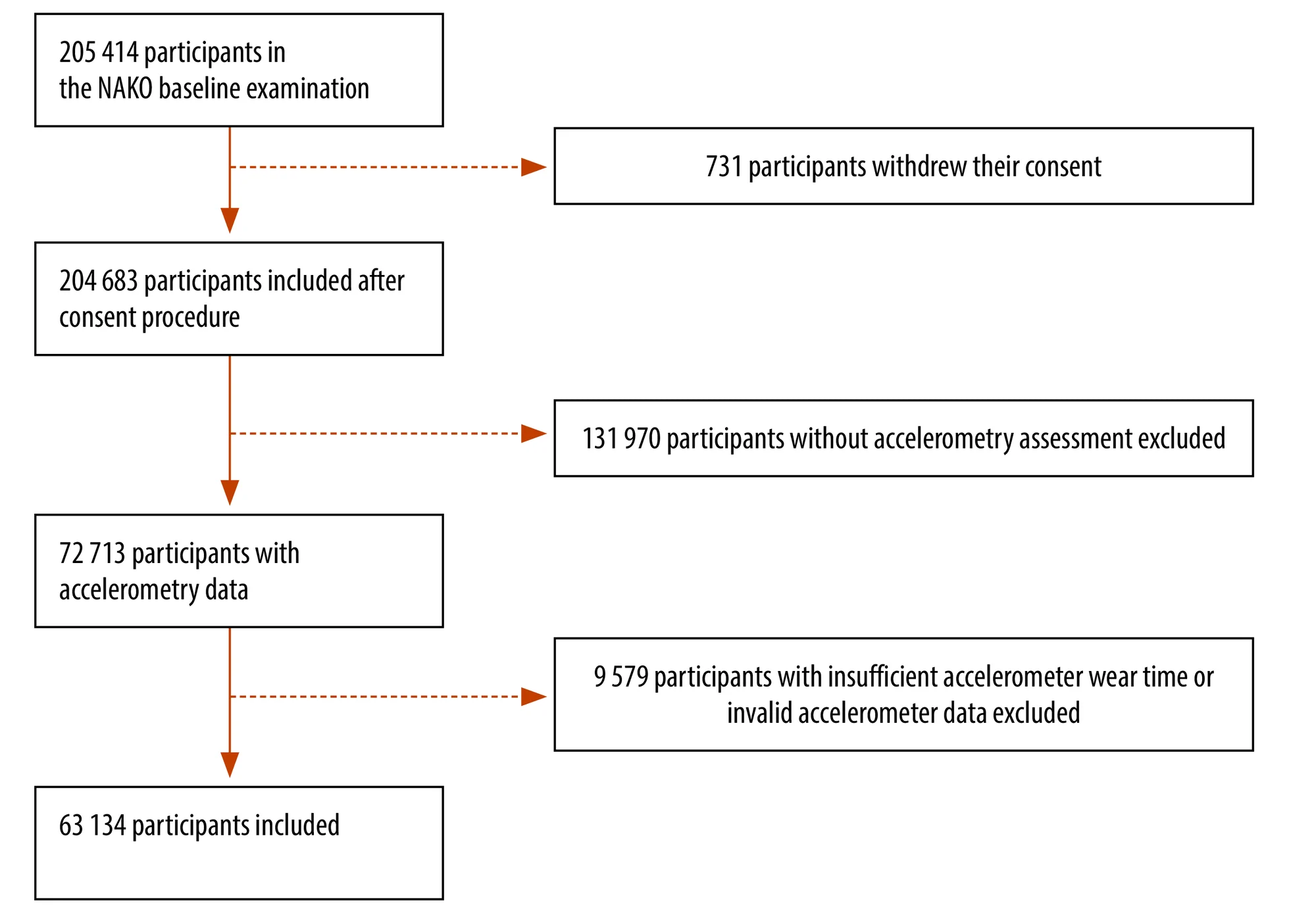
Participant flow in the study on the 10-minute bout requirement for physical activity guideline adherence, Germany, 2014–2019

NAKO: German National Cohort.

**Table 1:** Participant characteristics in the study on the 10-minute bout requirement for physical activity guideline adherence, Germany, 2014–2019

Characteristic	Total sample (*n* = 63 134)	Adherents (*n* = 22 226)	Transitioners (*n* = 40 306)	Non-adherents (*n* = 602)	*P* for group difference^a^
**Sex, no. (%)**					< 0.001
Male	30 210 (47.9)	11 082 (49.9)	18 796 (46.6)	332 (55.1)	
Female	32 924 (52.1)	11 144 (50.1)	21 510 (53.4)	270 (44.9)	
**Age in years, no. (%)**					NA
19–29	5 678 (9.0)	2 681 (12.1)	2 980 (7.4)	17 (2.8)	
30–39	6 535 (10.4)	2 439 (11.0)	4 080 (10.1)	16 (2.7)	
40–49	16 533 (26.2)	5 933 (26.7)	10 566 (26.2)	34 (5.6)	
50–59	17 184 (27.2)	5 897 (26.5)	11 165 (27.7)	122 (20.3)	
60–69	15 895 (25.2)	4 885 (22.0)	10 663 (26.5)	347 (57.6)	
≥ 70	1 309 (2.1)	391 (1.8)	852 (2.1)	66 (11.0)	
**Age in years, mean (SD)**	50.1 (12.6)	48.6 (13.0)	50.8 (12.2)	60.8 (10.4)	< 0.001
**Education level, no. (%)^b^**					< 0.001
Low	1 553 (2.5)	365 (1.7)	1 142 (2.9)	46 (7.7)	
Medium	25 096 (40.6)	7 402 (34.4)	17 376 (43.8)	318 (53.4)	
High	35 152 (56.9)	13 775 (63.9)	21 145 (53.3)	232 (38.9)	
**BMI (kg/m2), no. (%)**					NA
< 18.5	674 (1.1)	268 (1.2)	399 (1.0)	7 (1.2)	
≥ 18.5 and < 25.0	26 567 (42.1)	11 484 (51.7)	14 953 (37.1)	130 (21.6)	
≥ 25.0 and < 30.0	23 454 (37.2)	7 971 (35.9)	15 310 (38.0)	173 (28.7)	
≥ 30.0 and < 35.0	8 816 (14.0)	2 021 (9.1)	6 651 (16.5)	144 (23.9)	
≥ 35.0 and < 40.0	2 611 (4.1)	388 (1.7)	2 135 (5.3)	88 (14.6)	
≥ 40.0	994 (1.6)	87 (0.4)	847 (2.1)	60 (10.0)	
**BMI in kg/m2, mean (SD)**	26.4 (4.8)	25.2 (4.0)	27.0 (5.1)	30.6 (7.1)	< 0.001
**Self-reported overall health, no. (%)**					NA
Poor	459 (0.7)	94 (0.4)	324 (0.8)	41 (7.3)	
Less good	5 765 (9.3)	1 346 (6.2)	4 221 (10.7)	198 (35.1)	
Good	34 065 (55.2)	10 700 (49.1)	23 083 (58.6)	282 (50.0)	
Very good	19 066 (30.9)	8 387 (38.5)	10 642 (27.0)	37 (6.6)	
Excellent	2 349 (3.8)	1 246 (5.7)	1 097 (2.8)	6 (1.1)	
**Self-reported overall health score, mean (SD)**	3.3 (0.7)	3.4 (0.7)	3.2 (0.7)	2.6 (0.8)	< 0.001

BMI: body mass index; NA: not applicable; SD: standard deviation.

^a^ Refers to differences among the three groups: Adherents, transitioners and non-adherents

^b^ Classified using the International Standard Classification of Education 2011 level^24^ and categorized as low (primary and lower-secondary education), medium (upper-secondary and post-secondary, non-tertiary education) or high (bachelor’s or equivalent level and above).

Notes: adherents are those who met the physical activity guideline with the bout (participants in this group automatically also met the physical activity guideline without the bout); transitioners are those who did not meet the guideline with the bout, but met the guideline without the bout. Non-adherents are those who did not meet the guideline without the bout and therefore met neither guideline. Due to missing data, the number of participants in the variables education level, BMI and self-reported overall health does not equal the total sample size reported for each column.

### Guideline adherence

Among participants, 35.2% (22 226) were adherents who met the guideline with the bout, an additional 63.8% (40 306) of participants qualified as transitioners, which together resulted in 99.0% (62 532) meeting the guideline without the bout. Only 1.0% (602) of participants were in the non-adherents category, not meeting either guideline.

The sensitivity analyses using ± 10 milligravity as the acceleration thresholds for classifying moderate and vigorous intensity activity showed that, for activity without the bout, 98.3% (62 065) met the guideline at the higher thresholds and 99.5% (62 835) at the lower thresholds. For the activity with the bout, 31.7% (20 045) met the guideline at the higher thresholds and 39.5% (24 934) at the lower thresholds.

### Physical activity volumes

Weekly volumes of both moderate and vigorous activity were considerably higher when activity without the bout was included than when only the activity with the bout was considered (Table 2). Under both conditions, participants accumulated substantially more moderate activity than vigorous activity.

**Table 2:** Participant weekly activity levels in the study on the 10-minute bout requirement for physical activity guideline adherence, Germany, 2014–2019

Intensity of physical activity	Mean minutes per week (SD)				*P* for group difference^a^
	Total sample (*n* = 63 134)	Adherents (*n* = 22 226)	Transitioners (*n* = 40 306)	Non-adherents (*n* = 602)	
**With bout **					
Moderate	123.5 (133.3)	256.4 (140.3)	52.0 (43.9)	6.6 (15.1)	< 0.001
Vigorous	9.4 (32.6)	24.6 (50.9)	1.1 (6.0)	0.1 (1.0)	< 0.001
Moderate and vigorous	132.9 (141.3)	280.9 (138.9)	53.1 (44.1)	6.7 (15.2)	NA
**Without bout**					
Moderate	565.9 (216.4)	695.5 (205.4)	501.2 (183.0)	108.5 (34.3)	< 0.001
Vigorous	18.5 (39.3)	40.0 (57.9)	6.9 (13.2)	0.3 (1.7)	< 0.001
Moderate and vigorous	584.3 (226.6)	735.5 (209.0)	508.1 (185.9)	108.8 (34.2)	NA

NA: not applicable; SD: standard deviation.

^a^ Refers to differences among the three groups: adherents, transitioners and non-adherents.

Notes: adherents are those who met the physical activity guideline with the bout (participants in this group automatically also met the physical activity guideline without the bout); transitioners are those who did not meet the guideline with the bout but met the guideline without the bout. Non-adherents are those who did not meet the guideline without the bout and therefore met neither guideline. Moderate and vigorous physical activity was calculated by summing moderate and physical activity. Note that means and standard deviations are reported to facilitate comparability and interpretability, despite right-skewness in several variables.

Activity volumes were highest among adherents, intermediate among transitioners and lowest among non-adherents under both definitions. Fig. 2 and Fig. 3 visualize the respective moderate and vigorous activity volumes, respectively.

**Fig. 2:**
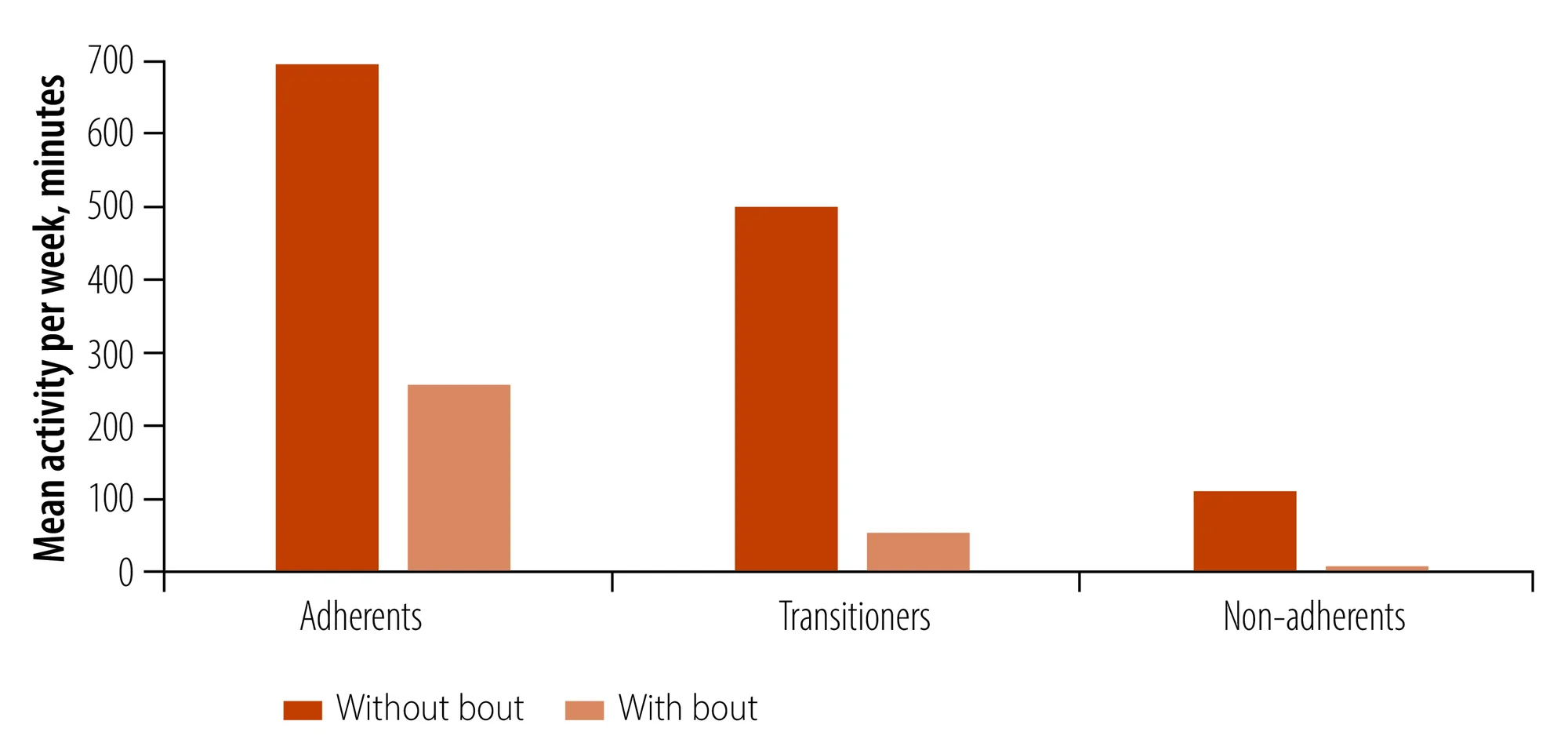
Mean weekly volumes of moderate physical activity in the study on the 10-minute bout requirement for physical activity guideline adherence, Germany, 2014–2019

Note: acceleration thresholds used were 90 milligravity for moderate physical activity. All group differences and pairwise comparisons reached statistical significance (*P*-values: < 0.001).

**Fig. 3:**
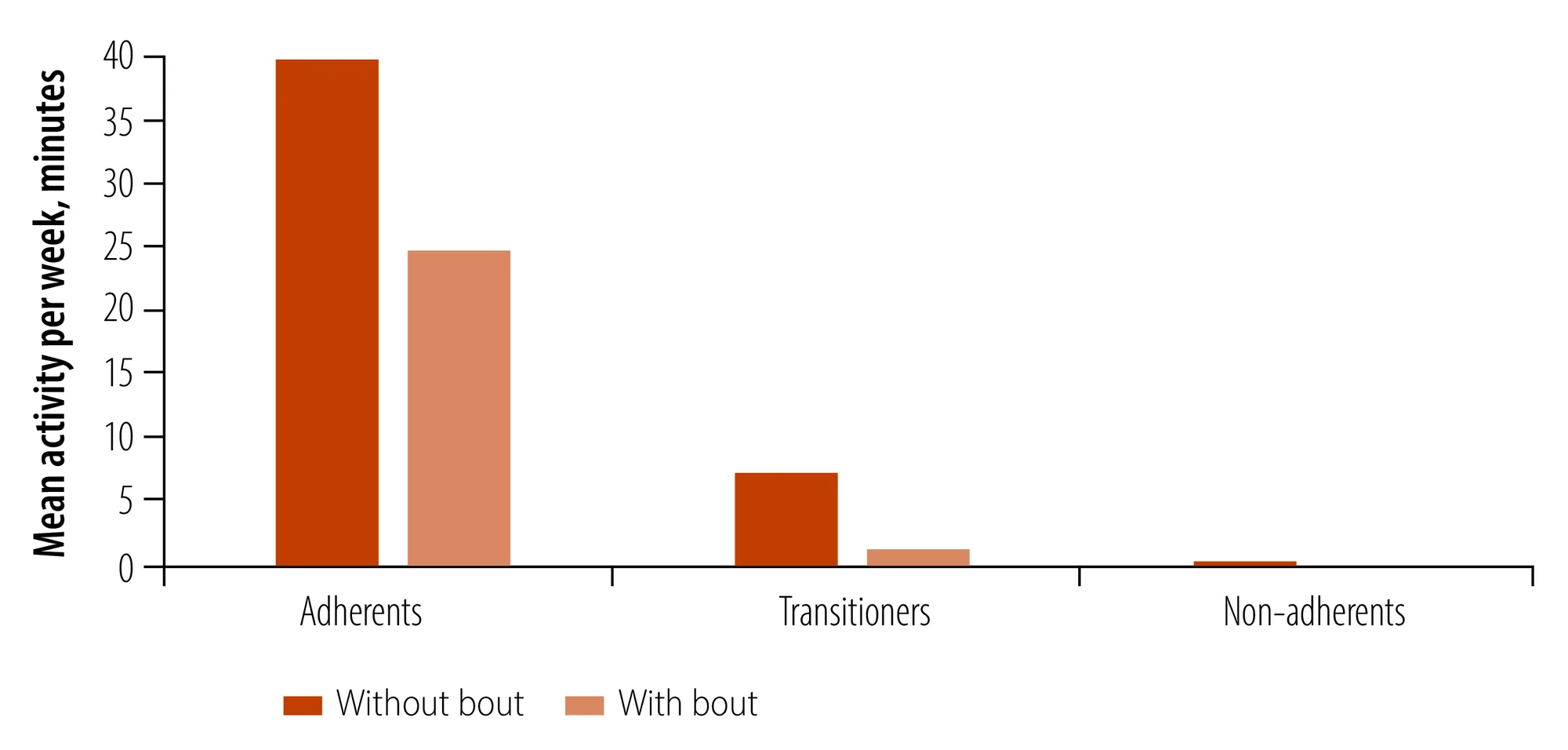
Mean weekly volumes of vigorous physical activity in the study on the 10-minute bout requirement for physical activity guideline adherence, Germany, 2014–2019

Note: acceleration thresholds used were 410 milligravity for vigorous physical activity. All group differences and pairwise comparisons reached statistical significance (*P*-values: < 0.001).

### Associated factors

The demographic and health-related characteristics differed across adherents, transitioners and non-adherents (Table 1). Analysis of variance with post-hoc pairwise comparisons yielded statistically significant results (all P-values: < 0.001) showing that adherents were younger, had the lowest BMI, and reported better overall health than the other two groups. The differences were most pronounced in the comparisons between adherents and non-adherents: On average, the adherents were 12.1 years (95% CI: 11.1–13.2) younger, their BMI was 5.5 kg/m2 (95% CI: 4.8–6.1) lower, and their self-reported overall health was 0.8 points (95% CI: 0.8–0.9) higher. Transitioners were located in between the adherents and the non-adherents for all three measures. Compared with adherents, transitioners were on average 2.1 years (95% CI: 1.9–2.4) older, had a BMI 1.9 kg/m2 (95% CI: 1.8–1.9) higher and reported overall health scores 0.2 points (95% CI: 0.2–0.2) lower. Compared with the non-adherents, transitioners were 10.0 years (95% CI: 9.0–11.0) younger, had a BMI that was 3.6 kg/m2 (95% CI: 2.9–4.3) lower, and their self-reported overall health was 0.6 points (95% CI: 0.5–0.7) higher.

The tests of equal proportions revealed statistically significant differences in the sex and education composition of the groups (Table 1). The post-hoc tests showed several statistically significant pairwise comparisons. There was a greater proportion of females among the transitioners than among the adherents and the non-adherents (all P-values: < 0.001). While, among the adherents and the transitioners, there was a higher proportion of participants with high versus medium education, among the non-adherents, there was a greater proportion of participants with medium versus high education (all P-values: < 0.001).

## Discussion

Based on accelerometry data in a large population-based sample from Germany, our study found that removing the 10-minute bout requirement nearly tripled adherence to WHO aerobic physical activity guidelines. Further, there were systematic differences in demographic and health-related characteristics among participants meeting the guidelines when the 10-minute bout requirement was removed rather than applied.

The finding that guideline adherence was notably higher when using activity without the bout is consistent with previous studies examining the impact of the bout requirement on accelerometry-measured guideline adherence.^11^^–^^13^ The findings appear plausible, as the removal of the bout requirement considerably broadens the range of physical activity that counts towards guideline adherence. Physical activity that is now considered guideline-relevant includes brief forms of exercise and non-exercise activities. Brief forms of physical exercise, such as high-intensity interval training, have well documented health benefits, including reducing adiposity and increasing cardiorespiratory fitness.^27^^,^^28^ A growing body of evidence also indicates that short bursts of exercise and intermittent physical activity can promote health.^29^^,^^30^

It is important to acknowledge that removing the 10-minute bout requirement hampers the analysis of time trends in moderate and vigorous physical activity guideline adherence. Increases in adherence are partly due to changes in the guidelines rather than actual increases in physical activity. Therefore, accelerometry-based studies examining guideline adherence should report results with and without the bout to enable comparisons with previous studies.

Our study also identified clear differences in the demographic, health and physical activity characteristics among individuals meeting the moderate and vigorous physical activity guidelines. Overall, we observed a gradient: those meeting the guideline with the bout were the youngest, had the lowest BMI, reported the best health and accumulated the most moderate and vigorous physical activity. Differences by sex and education were also evident. Taken together, and contrary to a previous accelerometry study,^11^ we found that the revised guidelines activity without the bout seem to capture a different demographic than the guidelines with the bout, although it should be noted that almost all participants were classified as adhering to the guideline of activity without the bout. The small subset of the sample that still did not meet the broader guidelines may represent an important target for public health interventions. Interestingly, despite this small subset of participants not meeting the guideline without the bout, around 10% of the total sample (6224/61 704 with data on self-reported overall health) reported less-than-good or poor overall health. This finding suggests that the guideline without the bout targets might be so low that individuals with health impairments can still meet them.

Several lessons emerge from our study. First, the revisions to the 2020 WHO guidelines relied on self-reported physical activity data, which may not translate well to accelerometry-derived metrics due to discrepancies between measures.^31^ Future guidelines should incorporate more evidence from accelerometry-based assessments.^10^^,^^32^^,^^33^ Notably, almost all participants met the guideline when the 10-minute bout requirement was removed, raising questions about whether most individuals genuinely achieve sufficient moderate and vigorous physical activity levels or if the thresholds need to be reconsidered. Second, methodological choices in accelerometry research, such as epoch length, substantially influence activity estimates. Short epochs capture brief, sporadic movements prompting questions of whether higher activity thresholds may be necessary or the use of longer epochs for comparability with existing evidence. Harmonization efforts are needed to address processing differences while maintaining clarity and usability for the general population.

A strength of our study is the use of a large, population-based sample, aiding subgroup analysis and the use of accelerometry to overcome self-report bias. Accelerometry may be particularly valuable for assessing activity without the bout by helping to overcome individuals’ recall issues, such as forgetting brief and irregular bursts of activity. However, our approach also has limitations. The sampling strategy may have introduced bias due to differences between study centres and self-selection bias of participants willing to engage in the accelerometry module. Similarly, the baseline data of the NAKO study, collected between 2014 and 2019, may not fully reflect current activity patterns in Germany given the widespread use of device-based self-monitoring of activity. Further, accelerometry analysis involves certain limitations related to computational approaches. We required participants to have valid data for at least two weekdays and one weekend day to better represent weekly activity, considering the typical differences in activity patterns between weekdays and weekends.^34^^,^^35^ Our method averaged data across available days and extrapolated to a full week, implicitly assuming that the observed days are representative of the entire period. Further potential concerns pertain to differences in results due to analysis choices^36^^,^^37^ or the generalizability of thresholds derived from smaller studies.^21^ We have documented our analytical approach to facilitate future evaluations and replication. Our sensitivity analyses using several acceleration thresholds yielded consistent results, but limitations like reactivity bias, where participants may move more due to wearing the accelerometer, cannot be completely ruled out.

In conclusions, our study found that applying the revised guidelines to accelerometry data substantially changes estimates of population adherence and the characteristics of those classified as meeting the guidelines. The findings have important implications for the interpretation of time trends in physical activity guideline adherence, the identification of which population subgroups are meeting or are not meeting the guidelines, and methodological considerations for analysing accelerometry data for guideline development.
